# Supplementary material for: A Translational Mouse Model for NASH with Advanced Fibrosis and Atherosclerosis Expressing Key Pathways of Human Pathology
Source: Cells. 2020 Sep 1;9(9):2014. doi: 10.3390/cells9092014 (PMC7565967; doi:10.3390/cells9092014)
Supplement: Supplementary file 1 [file cells-09-02014-s001.pdf]

Appendix A

Supplemental figures and table

(**a**)

Moylan fibrosis gene set

HFD vs. chow	FFD vs. chow	
2.2	3.5	COL1A1
2.0	3.2	COL3A1
2.1	3.1	COL1A2
1.8	2.9	CXCL6
1.9	2.6	VCAN
1.5	2.6	EHF
1.2	2.6	THBS2
1.3	2.4	COL6A3
1.9	2.4	LUM
1.2	2.4	COL4A1
0.9	2.4	FBN1
1.4	2.3	SOX4
1.6	2.1	ITGBL1
1.3	2.0	DPT
1.2	2.0	EPCAM
1.0	2.0	DKK3
1.1	1.9	BICC1
1.4	1.9	FLRT2
1.4	1.8	LAMA2
0.8	1.8	LBH
1.1	1.8	SRPX
1.2	1.7	FSTL1
1.3	1.7	EPHA3
0.8	1.6	COL14A1
0.5	1.5	DCDC2
0.5	1.5	C7
0.7	1.4	ANTXR1
0.7	1.4	EFEMP2
0.9	1.2	GLS
0.4	1.2	SOX9
0.6	1.2	GLT8D2
1.0	1.1	FBLN5
0.6	1.1	JAG1
0.9	1.1	MGP
0.6	1.1	IGFBP7
0.7	1.0	BCL2
0.8	0.8	CCDC146
0.4	0.7	STMN2
0.7	0.7	PDGFD
1.3	0.7	NALCN
0.0	0.7	TAX1BP3
0.6	0.6	MAP1B
0.2	0.6	ID4
0.5	0.6	EFEMP1
0.8	0.6	DCN
0.3	0.5	MSRB3
0.2	0.4	LIMA1
-0.6	0.4	AQP1
0.0	0.4	TAGLN
-0.3	0.1	C1orf198
-0.1	0.1	CLDN11
0.2	0.1	GEM
0.8	-0.1	PLCXD3
-1.5	-0.2	Fxyd2
0.9	-0.2	CHST9
-0.6	-0.3	CLDN10
-0.3	-0.3	PNMA1
0.2	-0.5	NEXN
0.2	-0.6	ANK3
-1.3	-0.8	CYBRD1

Supplemental Figure S1. Heatmap showing recapitulation of hepatic gene expression profile that differentiates NASH patients with mild fibrosis (stage F0 or 1) from severe fibrosis (stage F3 or 4) [27]

in Ldlr-/-.Leiden mice fed the HFD or FFD for 28 weeks relative to chow. Blue colour indicates down-regulation and red colour indicates upregulation. N ≥8 mice per group.

(a).

Representation of human NASH pathways:
**HFD & FFD:**
Actin Cytoskeleton Signaling
Atherosclerosis Signaling
Axonal Guidance Signaling
B Cell Receptor Signaling
Clathrin-mediated Endocytosis Signaling
Complement System
Dendritic Cell Maturation
Endothelin-1 Signaling
Ephrin A Signaling
Ephrin Receptor Signaling
Fc Epsilon RI Signaling
Fcγ Receptor-mediated Phagocytosis in Macrophages and Monocytes
FXR/RXR Activation
Germ Cell-Sertoli Cell Junction Signaling
G-Protein Coupled Receptor Signaling
Gαq Signaling
Hepatic Cholestasis
Hepatic Fibrosis / Hepatic Stellate Cell Activation
iCOS-iCOSL Signaling in T Helper Cells
IL-8 Signaling
Integrin Signaling
Leukocyte Extravasation Signaling
LPS-stimulated MAPK Signaling
LXR/RXR Activation
Macropinocytosis Signaling
mTOR Signaling
Natural Killer Cell Signaling
NGF Signaling
p70S6K Signaling
Phagosome Formation
Phospholipase C Signaling
PI3K Signaling in B Lymphocytes
PPARα/RXRα Activation
Production of Nitric Oxide and Reactive Oxygen Species in Macrophages
Regulation of Actin-based Motility by Rho
RhoA Signaling
RhoGDI Signaling
Role of Macrophages, Fibroblasts and Endothelial Cells in Rheumatoid Arthritis
Role of Osteoblasts, Osteoclasts and Chondrocytes in Rheumatoid Arthritis
Sphingosine-1-phosphate Signaling
Superpathway of Inositol Phosphate Compounds
Thrombin Signaling
Type II Diabetes Mellitus Signaling
Virus Entry via Endocytic Pathways
**FFD only:**
Actin Nucleation by ARP-WASP Complex
Ephrin B Signaling
ERK/MAPK Signaling
G Beta Gamma Signaling
Growth Hormone Signaling
Histidine Degradation VI
Role of NFAT in Cardiac Hypertrophy
Semaphorin Signaling in Neurons
**HFD only:**
Epithelial Adherens Junction Signaling
Extrinsic Prothrombin Activation Pathway
VDR/RXR Activation
**Not represented:**
Cell Cycle Control of Chromosomal Replication
Chemokine Signaling
Cysteine Biosynthesis III (mammalia)
D-myo-inositol (1,4,5)-Trisphosphate Biosynthesis
Glycerol-3-phosphate Shuttle
Intrinsic Prothrombin Activation Pathway
Sphingomyelin Metabolism
Superpathway of Methionine Degradation
Synaptic Long Term Depression
UVC-Induced MAPK Signaling

Supplemental Figure S2. List of differentially expressed pathways distinguishing human NASH patients vs. normal controls [6] and representation thereof in Ldlr-/-.Leiden mice fed the HFD or FFD for 28 weeks. N ≥8 mice per group.

Table S1. Metabolic parameters in time.

**Glucose (mM)**	**t = 0**	**t = 6**	**t = 12**	**t = 18**	**t = 22**	**t = 28**
Chow	7.4 ± 0.2	7.5 ± 0.3	6.6 ± 0.4	7.7 ± 0.4	7.7 ± 0.4	7.7 ± 0.4
HFD	7.1 ± 0.2	8.4 ± 0.6*	9.0 ± 0.6**	8.2 ± 0.5	7.6 ± 0.1	7.7 ± 0.2
FFD	7.4 ± 0.2	6.5 ± 0.2	6.6 ± 0.3	6.2 ± 0.2**	5.8 ± 0.2**	6.5 ± 0.3**
**Insulin (ng/mL)**	**t=0**	**t=6**	**t=12**	**t=18**	**t=22**	**t=28**
Chow	1.0 ± 0.2	1.1 ± 0.2	1.0 ± 0.2	2.0 ± 0.3	2.2 ± 0.4	2.9 ± 0.6
HFD	2.3 ± 1.3	5.5 ± 0.9***	9.8 ± 2.1***	12.1 ± 1.8**	14.7 ± 2.6***	14.7 ± 4.2***
FFD	1.7 ± 0.4	3.6 ± 1.0***	4.1 ± 1.3**	5.7 ± 0.4***	4.7 ± 0.5***	3.9 ± 0.4
**Cholesterol (mM)**	**t=0**	**t=6**	**t=12**	**t=18**	**t=22**	**t=28**
Chow	6.2 ± 0.3	8.7 ± 0.7	10.5 ± 1.0	10.3 ± 0.6	9.8 ± 0.8	8.0 ± 0.7
HFD	6.1 ± 0.7	20.6 ± 2.9*	28.4 ± 2.9**	27.5 ± 2.7**	39.4 ± 3.4***	32.2 ± 3.7***
FFD	6.6 ± 0.4	42.3 ± 3.8***	64.5 ± 6.3***	54.8 ± 4.6***	57.9 ± 5.3***	41.3 ± 4.7***
**Triglycerides (mM)**	**t=0**	**t=6**	**t=12**	**t=18**	**t=22**	**t=28**
Chow	1.4 ± 0.1	1.3 ± 0.2	2.2 ± 0.3	2.2 ± 0.2	2.0 ± 0.2	1.5 ± 0.3
HFD	1.2 ± 0.1	4.8 ± 0.9**	5.1 ± 0.7***	5.8 ± 0.8**	7.9 ± 1.1***	6.5 ± 1.3**
FFD	1.2 ± 0.1	9.4 ± 1.0***	13.5 ± 1.6***	13.5 ± 1.2***	13.8 ± 1.2***	8.3 ± 1.2***

Ldlr-/-.Leiden mice were fed a healthy chow diet or fed a high fat (HFD) diet containing lard fat or a fast food diet (FFD) containing milk fat for 28 weeks. Data represent mean ± SEM for n ≥8 mice /group. * $p < 0.05$, ** $p < 0.01$, *** $p < 0.001$ *vs.* chow.
